# Two Functional Motifs Define the Interaction, Internalization and Toxicity of the Cell-Penetrating Antifungal Peptide PAF26 on Fungal Cells

**DOI:** 10.1371/journal.pone.0054813

**Published:** 2013-01-21

**Authors:** Alberto Muñoz, Eleonora Harries, Adriana Contreras-Valenzuela, Lourdes Carmona, Nick D. Read, Jose F. Marcos

**Affiliations:** 1 Fungal Cell Biology Group, Institute of Cell Biology, University of Edinburgh, Edinburgh, United Kingdom; 2 Department of Food Science, Instituto de Agroquímica y Tecnología de Alimentos (IATA), Consejo Superior de Investigaciones Científicas (CSIC), Paterna, Valencia, Spain; Universidade de Sao Paulo, Brazil

## Abstract

The synthetic, cell penetrating hexapeptide PAF26 (RKKWFW) is antifungal at low micromolar concentrations and has been proposed as a model for cationic, cell-penetrating antifungal peptides. Its short amino acid sequence facilitates the analysis of its structure-activity relationships using the fungal models *Neurospora crassa* and *Saccharomyces cerevisiae*, and human and plant pathogens *Aspergillus fumigatus* and *Penicillium digitatum*, respectively. Previously, PAF26 at low fungicidal concentrations was shown to be endocytically internalized, accumulated in vacuoles and then actively transported into the cytoplasm where it exerts its antifungal activity. In the present study, two PAF26 derivatives, PAF95 (AAAWFW) and PAF96 (RKKAAA), were designed to characterize the roles of the N-terminal cationic and the C-terminal hydrophobic motifs in PAF26's mode-of-action. PAF95 and PAF96 exhibited substantially reduced antifungal activity against all the fungi analyzed. PAF96 localized to fungal cell envelopes and was not internalized by the fungi. In contrast, PAF95 was taken up into vacuoles of *N. crassa*, wherein it accumulated and was trapped without toxic effects. Also, the PAF26 resistant Δ*arg1* strain of *S. cerevisiae* exhibited increased PAF26 accumulation in vacuoles. Live-cell imaging of GFP-labelled nuclei in *A. fumigatus* showed that transport of PAF26 from the vacuole to the cytoplasm was followed by nuclear breakdown and dissolution. This work demonstrates that the amphipathic PAF26 possesses two distinct motifs that allow three stages in its antifungal action to be defined: (i) its interaction with the cell envelope; (ii) its internalization and transport to vacuoles mediated by the aromatic hydrophobic domain; and (iii) its transport from vacuoles to the cytoplasm. Significantly, cationic residues in PAF26 are important not only for the electrostatic attraction and interaction with the fungal cell but also for transport from the vacuole to the cytoplasm, which coincides with cell death. Peptide containment within vacuoles preserves fungal cells from peptide toxicity.

## Introduction

Antimicrobial peptides (AMPs) and proteins are being intensively studied as alternatives for the control of microorganisms in medical, agricultural and food preservation applications [1]–[4]. Nearly all AMPs are cationic and amphipathic molecules with the capability of interacting with and disrupting anionic biological membranes, which potentially enable them to permeabilize and lyse living microbial cells. In the last decade, however, it has become clear that some AMPs also have additional effects on their target microbial cells *in vivo*, including more subtle and specific intracellular mechanisms [5]–[11].

Various AMPs have been demonstrated to translocate across the plasma membrane non-disruptively at low to moderate concentrations and result in a variety of different intracellular effects [12]–[17]. AMPs with these characteristics share biophysical properties with the so-called cell penetrating peptides (CPPs). This has brought into question the differences between antimicrobial and cell penetrating activities [18] and led to the definition of a new class of cell penetrating AMPs (CP-AMPs) [4]. Peptides initially known as CPPs, and also found to be taken up into mammalian cells, were later demonstrated to exhibit antimicrobial activity towards distinct microorganisms, and this activity correlated with internalization of the CPPs by the microbial cells [19], [20]. However, the link between penetration and antimicrobial properties requires further analysis. Interestingly, for selected AMPs such as the proline-rich apidaecin, the microbial internalization and killing activities seem to be separated, and peptide uptake was necessary but not sufficient for antibacterial activity [12]. An attractive hypothesis, therefore, is that this class of bioactive peptides are in fact dual molecules in which internalization determinants do not necessarily overlap with antimicrobial activity [6]. Experiments should address the identification of these (separate) determinants in model AMPs in order to establish the minimum amino acid sequence requirements for these different activities, if present. This should help in the design of “modular” domains with distinct functional capabilities for custom-designing improved AMPs in the future.

PAF26 is a synthetic, tryptophan-rich, cationic hexapeptide that was previously identified by a combinatorial approach against the fungal plant pathogen *Penicillium digitatum* [21]. It has been shown to enter cells of *P. digitatum* and perturb cell morphogenesis prior to cell permeabilization, but it is not lytic or cytotoxic to human cells [22], [23]. Recently, PAF26 entry routes into fungal cells were analyzed in detail using live-cell imaging techniques in the model filamentous fungus *Neurospora crassa* [17]. This study demonstrated endocytic internalization of PAF26 prior to killing fungal cells, when the peptide was applied at low fungicidal concentrations. PAF26 is therefore a CP-AMP that possesses the determinants for both antifungal and cell penetrating activities in just six amino acid residues, and we have proposed it as a useful model to analyze these two activities as well as the relationships between them.

Treatment of *Saccharomyces cerevisiae* with sublethal concentrations of peptides demonstrated the differential effects of the CP-AMP PAF26 and the cytolytic peptide melittin at the transcriptional level [24]. This study showed increased expression of genes involved in strengthening of the cell wall (a common response to different AMPs), arginine metabolism, ribosomal biogenesis and the unfolded protein stress response [24]. Deletion of specific genes altered the sensitivity of the yeast to PAF26. For instance, deletion of the *ARG1* gene that encodes the cytosolic arginine succinate synthetase in the arginine biosynthetic pathway, as well as the deletion of other *ARG* genes, resulted in increased resistance to PAF26. Recently, increased endogenous nitric oxide (NO) production in the budding yeast was shown in response to PAF26 treatment and NO production correlated with peptide toxicity [25]. In addition, arginine-derived NO production was blocked in the Δ*arg1* mutant, providing a plausible explanation for its resistant phenotype.

In this study we took advantage of the small size and defined amino acid sequence of PAF26 to characterize the influence of the cationic N-terminal and the hydrophobic C-terminal motifs in its modes of internalization, intracellular transport and antifungal activity. By using PAF26 sequence analogs and analyzing their inhibitory activities and subcellular locations, we have defined three steps in PAF26 mode-of-action which are conserved in different fungi (the fungal models *Neurospora crassa* and *Saccharomyces cerevisiae*, and the human fungal pathogen *Aspergillus fumigatus*). We show that the peptide first encounters and interacts with the cell envelope (cell wall and/or the plasma membrane), subsequently enters the fungal cell, is transported to the vacuole, and finally exerts its killing activity after being transported to the cytoplasm.

## Results

### Both cationic and hydrophobic motifs are needed for PAF26 activity

The antifungal peptide PAF26 was identified through a synthetic combinatorial hexapeptide library screen against phytopathogenic fungi [21]. Its sequence is optimized in terms of antifungal potency, high selectivity for fungi [26] and lack of toxicity towards human cells [22], [23]. Interestingly, two motifs are present in the PAF26 sequence: a cationic N-terminus (RKK) and a C-terminus composed of hydrophobic, aromatic amino acids (WFW), which together conform an amphiphilic AMP (Table 1). In this study, we designed two derivatives of PAF26 to characterize the roles of these N- and C-terminal regions in its antifungal properties. PAF95 was designed to neutralize the strong cationic N-terminal half of the peptide by substituting its three residues with neutrally charged Ala residues. Similarly, the C-terminus was modified in PAF96 by replacing the hydrophobic amino acids with Ala residues. As expected, the isolelectric point and net charge of PAF95 were strongly reduced while the hydropathicity index (GRAVY) also changed significantly for both derivatives (Table 1).

**Table 1:** Peptides used in this study.

Peptide	Sequence	MW^a^	Net charge^a^	pI^a^	GRAVY^a^
PAF26	RKKWFW	950.1	+3	11.17	−1.883
PAF95	AAAWFW	750.8	0	5.57	1.067
PAF96	RKKAAA	643.7	+3	11.17	−1.150

a Calculated values according to ProtParam at the Expasy Server (www.expasy.org).

**Figure 1:**
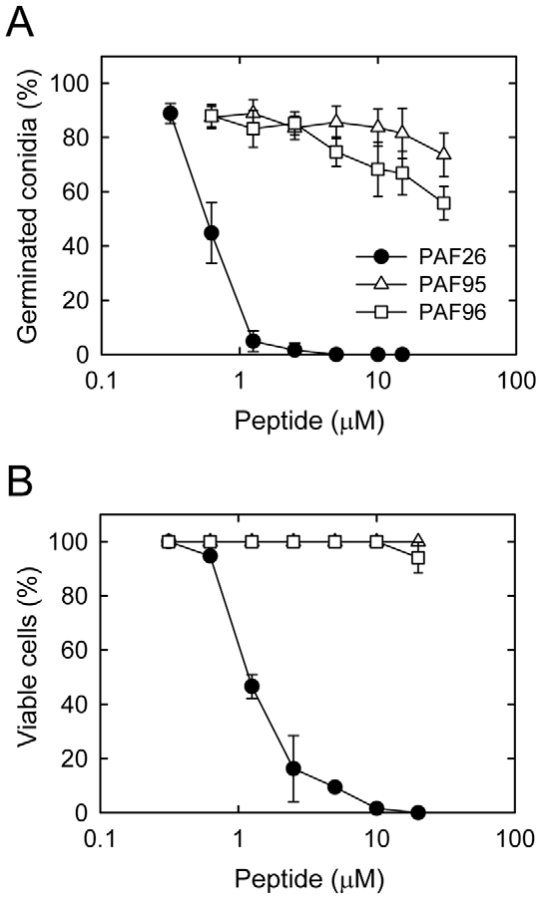
Dose-response curves of the effects of the PAF peptides on conidial germination and viability of conidia of *N. crassa*. (A) Percentage of germinated conidia was quantified by light microscopy 3 h after treatment with the peptides. (B) Percentage of viable cells after 3 h of peptide treatment was quantified using the cell death marker propidium iodide. The peptides used were PAF26 (black circles), PAF95 (white triangles) and PAF96 (white squares).

Following a previous analysis of the mechanisms of PAF26 cell penetration in the filamentous fungus *N. crassa* [17], we used this eukaryotic model to characterize the novel derivatives PAF95 and PAF96. A substantial reduction in the fungistatic and fungicidal activities of both new peptides in comparison with the parental peptide PAF26 was observed (Figure 1). Complete inhibition of conidial germination was achieved at ≥5 µM in the case of PAF26, whereas at a concentration of 30 µM, 74±8% and 56±6% germination were observed in the presence of PAF95 and PAF96, respectively (Figure 1A). Little or no killing of *N. crassa* conidia was observed after treatment with up to 20 µM of PAF95 or PAF96 (Figure 1B). This contrasted with the strong fungicidal activity of PAF26, which at 2.5 µM killed 81% of conidia.

### In *N. crassa* cells the interaction and localization of PAF peptides are dependent on specific amino acid motifs

Confocal microscopy of fluorescently labeled peptides has allowed the visualization of AMP internalization by microbial cells [13], [27]–[30], [17]. The PAF peptides were covalently labelled at their N-termini with the red fluorophore tetramethyl-rhodamine (TMR) as part of the synthetic procedure. Labeling of PAF26 with the TMR fluorescent label did not significantly influence the activity of PAF26 [17].

**Figure 2:**
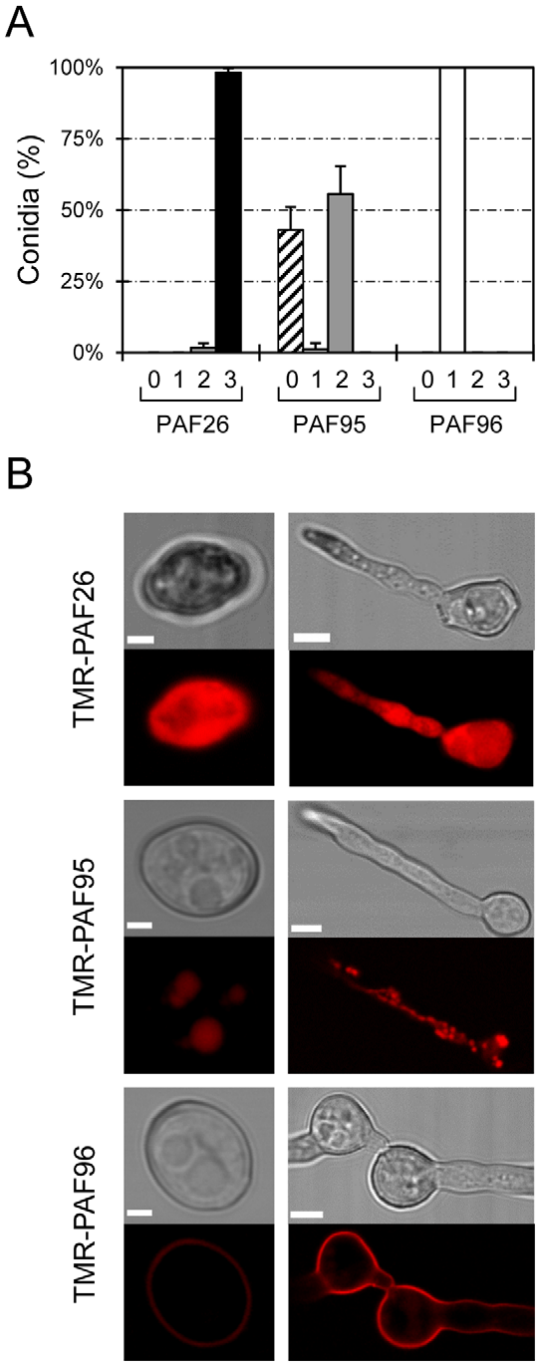
Localization of fluorescently labeled PAF peptides in *N. crassa* cells. (A) Percentage of conidial population that do not show any TMR-peptide fluorescence signal (class “0”, striped bars), or show TMR-peptide fluorescence limited to cell envelopes (class 1, white bars), intracellular organelles (class 2, light grey bars), or filling the whole cytoplasm of the cells (class 3, black bars), after 1 h of treatment with 5 µM of each of the TMR-peptides. (B) Representative confocal images showing the localization of TMR-PAF26, TMR-PAF95 and TMR-PAF96 (in red) in conidia (left panels) and conidial germlings (right panels). For each peptide, the most common pattern of localization is shown (see A). Bars: 2 µm in conidia and 5 µm in germlings.

The three peptides, each at a concentration of 5 µM exhibited different localization patterns with cells of *N. crassa* after 1 h of treatment (Figures 2 and S1). At this lethal peptide concentration (Fig. 1), TMR-PAF26 was detected throughout both conidia and germ tubes that became highly vacuolated and died (Figure 2B). Quantification revealed that 98±2% of the conidial population showed this characteristic staining pattern with TMR-PAF26 (Figure 2A). The non-active TMR-PAF95 that possessed the hydrophobic motif but lacked the cationic motif, was internalized by 56±10% of conidia. It accumulated within intracellular organelles that resembled the vacuolar system (see below) but did not stain the cell envelope or cytoplasm (Figure 2 and Figure S1). The remaining 43±8% of conidia showed no TMR-PAF95 labeling (Figure 2A). On the other hand, the non-active TMR-PAF96, that possesses the cationic but not hydrophobic motif, remained bound to the cell envelope (either or both the cell wall and the plasma membrane) and was not internalized by any of the conidia tested (Figure 2A, B).

**Figure 3:**
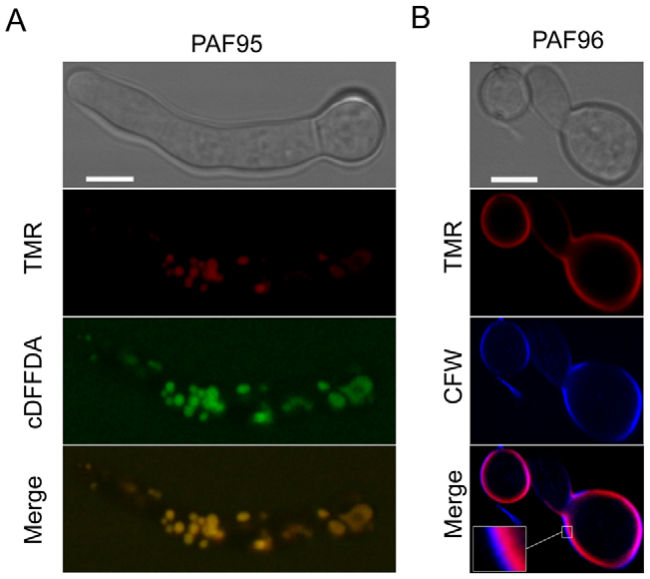
Subcellular localization of PAF95 and PAF96 in conidial germlings of *N. crassa*. (A) Co-localization of TMR-PAF95 (in red) with the vacuolar marker cDFFDA (in green) after treatment of the germlings with 5 µM TMR-PAF95 for 1 h. Note co-localization within the vacuolar network in these merged images. (B) Localization of TMR-PAF96 (in red) and the cell wall stain CFW (in blue) after treatment with TMR-PAF96 for 1 h. Note that in regions of the cell envelope the calcofluor white staining is exterior to that of the TMR-PAF96 labelling (see inset) in the merged image. Bar: 5 µm.

Further experiments were designed to better characterize the subcellular location of the TMR-labelled PAF95 and PAF96 peptides by using them in combination with other fluorescent reporters of cell walls and organelles. The vacuolar localization of TMR-PAF95 was confirmed by co-localizing the peptide with the vacuolar probe cDFFDA (Oregon Green 488 carboxylic acid) (Figure 3A). Staining the cell walls with the chitin binding probe calcofluor white (CFW) and co-labelling with TMR-PAF96 showed regions of the cell envelope where the staining of the cell wall was exterior to the TMR-peptide labeling (Figure 3B, see inset box). This indicates that the TMR-PAF26 will initially interact with the outer region of the cell wall (as it is the first encountered layer) and would subsequently reach the inner region of the cell wall and/or plasma membrane.

### PAF26 is fungicidal to and internalized by cells of the human pathogen *Aspergillus fumigatus*

**Figure 4:**
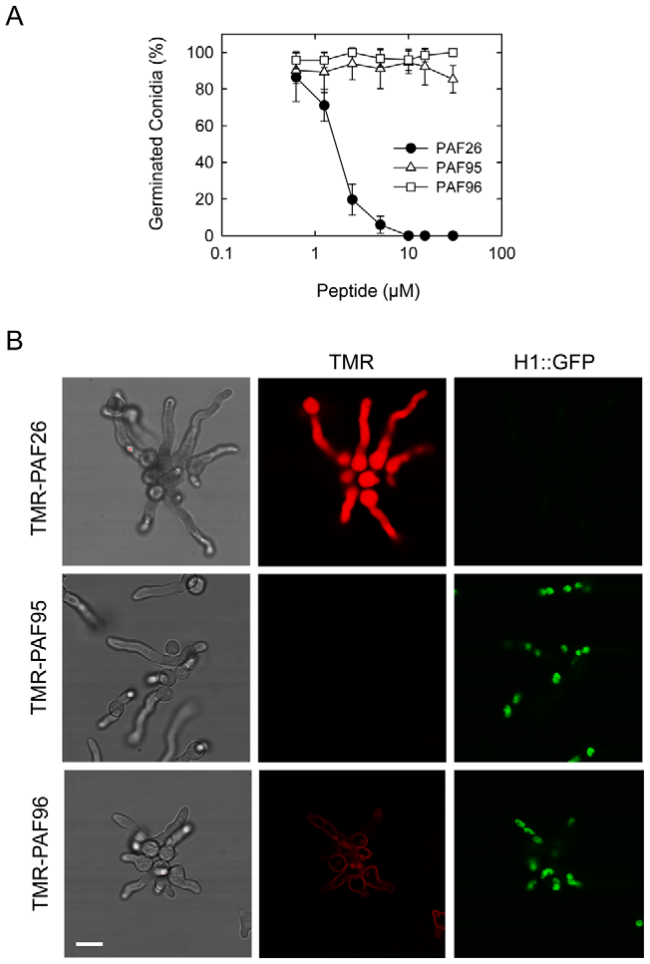
Activity and localization of PAF peptides in *A. fumigatus*. (A) Influence of PAF peptides on conidial germination. Percentage of germinated conidia was quantified by microscopy ∼5 h after treatment with the peptides at different concentrations. (B) Confocal microscopy of the localization of 5 µM TMR-labeled peptides (in red) in germlings of *A. fumigatus* expressing H1-GFP (in green) 1 h after treatment with the three peptides. Note that the left hand panels show brightfield images of germlings and the right panels show corresponding GFP-labeled nuclei in the same cells. The germlings were all treated for 1 h with the different PAF peptides before imaging. Note that the whole germlings treated with TMR-PAF26 are labeled by the fluorescent peptide, lack nuclei and are dead. This contrasts with both the TMR-PAF95 and TMR-PAF96 treated germlings that possess nuclei and are alive. The TMR-PAF95 treated germlings lack any labeling with the fluorescent peptide whilst TMR-PAF96 is bound only to the cell envelope of the germlings. Bar: 10 µm.

We also explored the use of PAF26 as a candidate AMP to control the human pathogen *A. fumigatus*. We tested the PAF26 inhibitory activity towards *A. fumigatus* in an assay in which ungerminated conidia were exposed to increasing concentrations of the peptide and incubated for 5 h. At 37°C in liquid Vogel's growth medium, 95% of untreated conidia underwent isotropic growth and germinated to produce germ tubes. A significant reduction in germination occurred when conidia were treated with 1–5 µM PAF26, while 10 µM PAF26 completely inhibited germination (Figure 4A). Similar to *N. crassa*, conidial germination in *A. fumigatus* was not significantly inhibited by the two PAF26 derivatives, PAF95 and PAF96 (Figure 4A).

**Figure 5:**
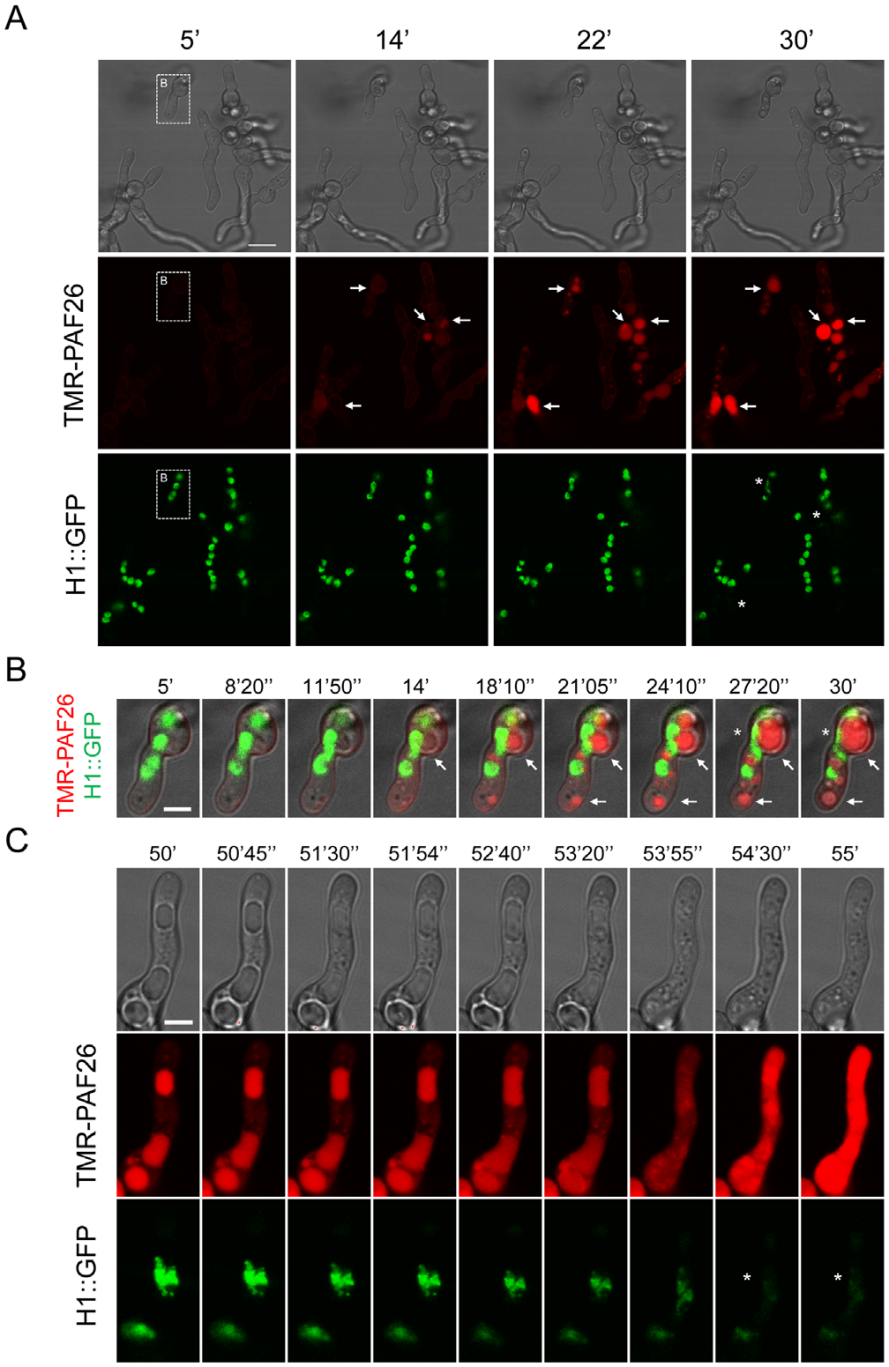
Interaction and internalization of TMR-PAF26 in *A. fumigatus*. (A) Sequential images show the interaction and internalization of 5 µM TMR-PAF26 (in red) in actively growing conidial germlings of *A. fumigatus* expressing H1-GFP (in green) over a period of 25 min. (B) Detail of a germling present in (A) showing merged fluorescent and brightfield images. Note accumulation of TMR-PAF26 in vacuolar compartments (arrows) which undergo expansion and become more intensely fluorescent with time. This coincided with the initiation of the breakdown and loss of fluorescence of the nuclei (asterisks). (C) Detailed images of a germling undergoing these effects over a period of 5 min recorded 50 min after peptide addition. See also Movie S1 for the entire sequential time course of panel (A). Bar: 10 µm in (A) and 4 µm in (B) and (C).

To characterize the interaction of PAF26 with conidial germlings of *A. fumigatus*, we exposed TMR-labelled peptides to a strain in which nuclei were labelled with GFP fused to the histone H1 (Figures 4B and 5). After incubation for 1 h with each of the peptides at a concentration of 5 µM, only the germlings treated with TMR-PAF26 were uniformly fluorescently stained throughout and were dead. Consistent with this, ≥95% of TMR-PAF26 treated germlings lacked any nuclear staining whilst those treated with TMR-PAF95 or TMR-PAF96 exhibited normal GFP nuclear labeling providing further evidence of PAF95 and PAF96 lacking antifungal activity (Figure 4B). Similar evidence of the nuclear breakdown coincident with a cytosolic PAF26 signal was also reported previously in a *N. crassa* GFP-H1 strain [17]. However, in marked contrast to what occurs in *N. crassa* (Figures 2B and 3A), we could not detect any signal of the inactive TMR-PAF95 within conidia or germ tubes of *A. fumigatus*. In *N. crassa*, TMR-PAF95 penetrated and was compartmentalized within the vacuolar network of 56±10% of cells (Figure 2A). This suggests significant differences with regard to the interaction of PAF95 with *N. crassa* and *A. fumigatus* germlings. In the former, the inactive peptide was able to interact and penetrate a proportion of the cell population while in the latter (Figure 5B) no interaction of PAF95 with cells could be observed. On the other hand, PAF96 behaved similarly in both fungi being located at the cell envelope with no evidence of cell penetration (compare Figures 2B and 4B).

Germinated and ungerminated conidia were treated with inhibitory concentrations of TMR-PAF26 and analyzed by time-lapse confocal microscopy. After 5–14 min of exposure to TMR-PAF26, faint red fluorescence indicative of peptide internalization were detected within spherical vacuoles which increased in size and intensity between 14 and 30 min (see arrows in Figure 5A and B). A total absence of co-localization between H1-GFP and TMR-PAF26 was observed. Towards the end of this 30 min period the green nuclear fluorescence in some of the nuclei started to disappear (see asterisks in Figure 5, Movie S1), indicating chromatin dissolution and probably cell death. At longer times of peptide treatment (∼1 h), TMR-PAF26 was seen to be transported from the vacuoles to the cytosol resulting in intense staining throughout the cells, and this was associated with the breakdown and dissolution of the nuclei and death of all of the cells (see PAF26 panel in Figure 4B and time course of Figure 5C).

### The unicellular model fungus *Saccharomyces cerevisiae* also shows differential susceptibility and interaction with the PAF peptides

**Figure 6:**
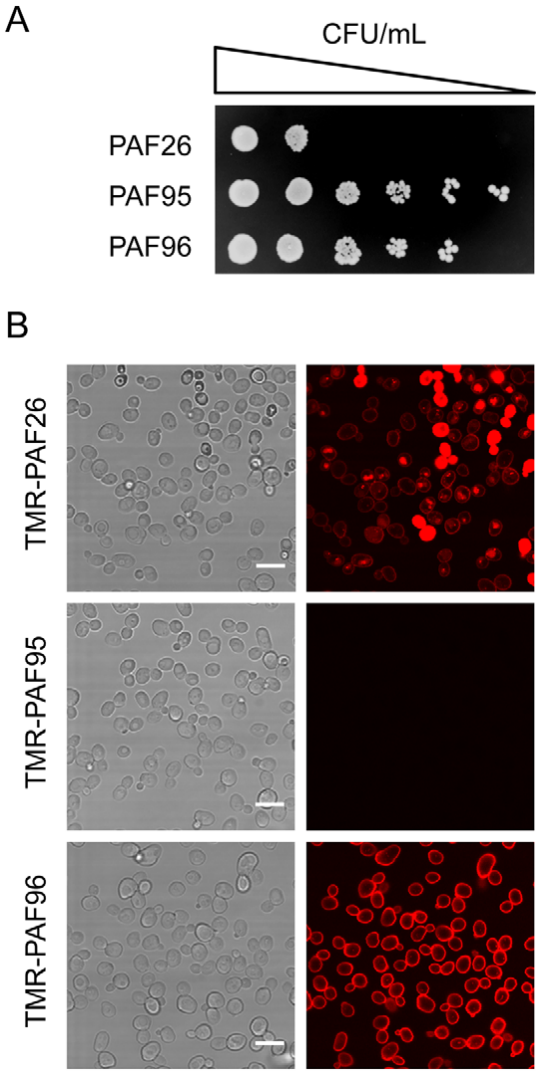
Activity and localization of PAF peptides in yeast. (A) Serial 5-fold dilutions of exponentially growing *S. cerevisiae* BY4741 cells treated with 64 µM of each peptide for 24 h, and subsequently plated onto YPD peptide-free plates. (B) Brightfield (images on the left) and corresponding confocal microscopy (images on the right) showing the localization in yeast cells (1×10^6^ cells/ml) after treatment with 2.5 µM of each of the TMR-labeled peptides (in red) for 30 min. Bar: 10 µm.

We have previously reported that PAF26 shows antifungal activity against *S. cerevisiae* and that FITC-PAF26 is internalized by yeasts cells [24]. Similar to the results reported above for *N. crassa* and *A. fumigatus*, PAF95 and PAF96 showed a very significant reduction in antifungal activity against *S. cerevisiae* compared with that of PAF26 (Figure 6A).

**Figure 7:**
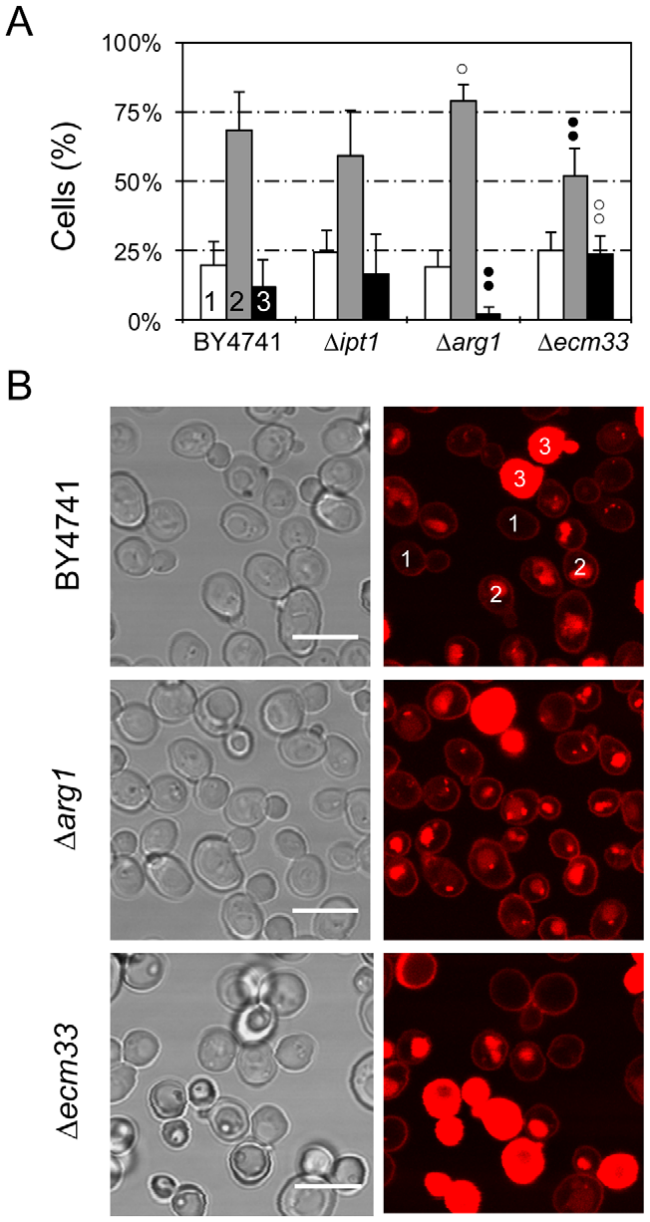
Differential localization of TMR-PAF26 in different *S. cerevisiae* strains. (A) Graph showing the percentage of cells that show TMR-PAF26 fluorescence limited to the cell envelope (class 1, white bars), in intracellular organelles (class 2, light grey bars), or filling whole cells (class 3, black bars), after treatment for 1 h with the peptide for each strain (as labeled on the x-axis). Cell location was determined by confocal microscopy. Results represent the means ± standard deviations of three experiments, each performed in triplicate. Dots above bars indicate statistical significance as compared to each class in the BY4741 parental strain control. Black dots indicate percentage lower than BY4741 and white dots higher. Two dots indicate p<0.01, and one dot p<0.05. (B) Representative confocal microscopy images are shown from the data set quantified in (A). Two examples of classes 1, 2 and 3 are labelled in strain BY4741. Bar: 10 µm.

Confocal microscopy showed that TMR-PAF26 exhibited different patterns of staining within the population of yeast cells of the reference strain BY4741 (Figure 6B). With 2.5 µM TMR-PAF26, 15–25% of cells had only their cell envelopes labeled, 60–75% showed labeling of their cell envelopes and also discrete intracellular vacuole-like structures, and 3–20% exhibit intense red staining throughout the cell and this correlated with cell death (Figure 7A). Localization of TMR-PAF26 within vacuole-like structures was coincident with that of the vacuolar probe cDFFDA (data not shown). TMR-PAF95 lacking the cationic motif did not stain these wild type cells at all, similar to the result obtained with *A. fumigatus* (Figure 4B), and TMR-PAF96 that lacked the hydrophobic motif exclusively labeled the cell envelope of yeast cells (Figure 6B). When TMR-PAF95 and TMR-PAF96 peptides were applied for longer times of incubation (i.e., 1 h, similarly to the *A. fumigatus* experiment in Figure 4B) and higher concentrations (up to 25 µM), the result was identical (data not shown) consistent with a complete lack of interaction (PAF95) or internalization (PAF96) of these peptides.

A previous genomic approach identified *S. cerevisiae* mutants with specific gene deletions that showed altered sensitivities to PAF26 (Table 2) [24]. They correspond to: (1) *IPT1* that encodes a specific membrane sphingolipid; (2) several *ARG* genes from the urea cycle and metabolism of arginine (including *ARG1*); and (3) *ECM33*, which encodes a major cell wall GPI-anchored protein of unknown function. The mutant Δ*ecm33* possesses a weakened cell wall and shows increased sensitivity to PAF26. In contrast, gene deletions of either *IPT1* or *ARG1* resulted in increased resistance to PAF26 [24]. Interaction of TMR-PAF26 with the *S. cerevisiae* Δ*arg1* or Δ*ecm33* mutants showed a significantly different distribution of the TMR-PAF26 signal from that of the wild type BY4741 strain or Δ*ipt1* strain (Figure 7A). Representative micrographs are shown to illustrate that the TMR-PAF26 fluorescence detected in Δ*arg1* cells was mostly located in discrete vacuolar organelles and at a higher frequency than in BY4741 cells. In contrast, Δ*arg1* cells exhibiting TMR-PAF26 staining throughout their cells were scarce and this was coincident with the lower sensitivity of this strain to PAF26 (Figure 7B). On the other hand, the complete staining of cells with TMR-PAF26 in Δ*ecm33* cells was significantly more frequent (p<0.01) than the other strains, and this correlated with the higher sensitivity of this mutant to the peptide [24]. The three patterns of TMR-PAF26 distribution within cells of the Δ*ipt1* strain were not significantly different to that of the wild type BY4751 strain. Staining of the three mutant strains with TMR-PAF95 or TMR-PAF96 produced similar results to those obtained with BY4741 cells (data not shown).

**Table 2:** Saccharomyces cerevisiae gene deletion mutants used in this study.

Strain a	Genotype	Description b	PAF26 susceptibility c
BY4741	*MAT*a *his3*Δ*1 leu2*Δ0 *met15*Δ0 *ura*Δ*0*	Parental Strain	
Y04007	Same as BY4741 *ipt1*Δ::*KanMX4*	Inositolphosphotransferase, involved in synthesis of the sphingolipid mannose-(inositol-P)_2_-ceramide [M(IP)_2_C]	R
Y01750	Same as BY4741 *arg1*Δ::*KanMX4*	Arginosuccinate synthetase (arginine biosynthesis and urea cycle). Cytosolic.	R
Y03215	Same as BY4741 *ecm33*Δ::*KanMX4*	GPI-anchored protein of unknown function. Cell wall/plasma membrane.	S

a Strain name at the EUROSCARF collection (http://web.uni-frankfurt.de/fb15/mikro/euroscarf/index.html).

b Summary of annotations found at the Saccharomyces Genome Database (http://www.yeastgenome.org/).

c Susceptibility of each strain to PAF26, as reported in [24]. “R” stands for increased resistance and “S” for increased susceptibility, respectively, as compared to the parental strain BY4741.

## Discussion

We have used the synthetic PAF26 peptide as a model CP-AMP to analyze the determinants of both antifungal and cell penetrating activities of AMPs. This rationale is facilitated by: (i) its small size; (ii) use of sequence analogs of PAF26 that show different activities [23], [26]; and (iii) the availability of mutants of *S. cerevisiae* [24], [25] and *N. crassa* [17] that show altered susceptibility to the peptide. This study provides significant new evidence demonstrating the intracellular mode-of-action of PAF26. It also shows that interaction and internalization of the derivatives of PAF26 (i.e. PAF95 and PAF96) *per se* do not result in cell death. Our data clearly define three steps which are necessary but not sufficient for PAF26 fungicidal action: (1) a cationic motif-mediated interaction of the peptide with cell envelopes that is also influenced by fungal cell wall components; (2) tryptophan-mediated internalization; and (3) intracellular toxicity which is linked to the cationic charge of the peptide and active transport from the vacuoles into the cytosol.

In a previous study, an alanine scan experiment in which each amino acid of the PAF26 sequence was substituted by alanine was carried out to determine the contribution of each residue to the peptide's antimicrobial activity [26]. The main conclusion was that no single substitution is a determinant for activity, since all the residues contributed individually to some extent. It was also evident that any cationic amino acid (Arg, Lys) exhibits a quantitatively more important contribution than aromatic residues (Trp, Phe). As an alternative approach, two new hexapeptides were designed for the present study in which either the cationic domain (RKK) or hydrophobic domain (WFW) was removed and replaced with three alanine residues to produce the two PAF26 derivatives, PAF95 and PAF96, respectively. Our study clearly demonstrated that both domains are necessary for antifungal toxicity. Neither PAF95 nor PAF96 showed significant antifungal activity towards *N. crassa* (Figure 1), *A. fumigatus* (Figure 4A), *S. cerevisiae* (Figure 6A) or the plant pathogen *P. digitatum* (Figure S2). It is obvious that these substitutions greatly modified the physicochemical properties of the parental peptide PAF26 (Table 1), including the cationic charge that has been previously reported as critical for the activities of most AMPs [5], [7]–[10]. A substantial detrimental effect was thus anticipated. However, the different localization patterns of the three PAF peptides led to significant new conclusions on the roles of the two main domains of PAF26 in binding to the cell envelope, cell penetration, transport to the vacuole, transport out of the vacuole and its antifungal activity.

The use of the model filamentous fungus *N. crassa* represents a very amenable experimental system for which a broad range of live-cell imaging techniques are available [31], [32]. PAF26 was internalized by *N. crassa* germlings and eventually after long-term (1 h) exposure to the peptide it was localized throughout cells and this coincided with cell death (Figure 2B). A recent study has described in detail the kinetics and internalization mechanisms of PAF26 into this model fungus [17]. The mode of PAF26 uptake by cells was shown to be concentration dependent with endocytic internalization playing a predominant role at low fungicidal concentrations and passive translocation at higher concentrations. Endocytically internalized PAF26 accumulated in vacuoles and was then actively transported into the cytoplasm where it exerted its antifungal activity. In the present study, PAF95 and PAF96 showed distinctly different localization patterns to PAF26 within living cells of *N. crassa* (Figure 2), which demonstrated the different roles of the two PAF26 motifs during its dynamic internalization process.

*Neurospora crassa* was unique in that it was the only fungus out of the four analyzed in which TMR-PAF95 was internalized in a significant number of cells (50–60% of conidia, Figures 2A and S1). Co-localization with the vacuolar stain cDFFDA showed that PAF95 was localized in the vacuolar system (Figure 3). In the other three fungi (*S. cerevisiae*, *A. fumigatus* and *P. digitatum*), TMR-PAF95 was not internalized (Figures 4 and 6, and data not shown). Positive charge is recognized as a key driving force for AMP activity because it promotes interaction with negatively charged microbial surfaces, and our data confirm this for all the fungi analyzed. The behavior of PAF95 in *N. crassa* suggests that additional, unknown cell envelope factors exist in this fungus, which allow the interaction, penetration and transport of a non-cationic peptide as far as the vacuole, albeit with less efficiency than PAF26. We hypothesize that these uncharacterized fungal cell wall/plasma membrane factors that facilitate the interaction of PAF95 would: (i) interact with the aromatic tryptophan and/or phenylalanine residues in the hydrophobic domains of PAF26 and PAF95, and (ii) relate to the observed increased sensitivity and faster inhibitory responses of *N. crassa* exposed to PAF26, as compared to other fungi (compare Figures 1A, 4A, and S2). Factors that reside in the cell wall and/or the plasma membrane may not only facilitate the interaction of certain AMPs with the fungal cell but they could also mask receptors/target molecules that the AMPs may interact with in this cell region. Examples of possible ‘masking’ molecules include cell wall glycoproteins that increase the resistance of *Fusarium oxysporum* to osmotin [33] and stress proteins of the PIR family that reduce the inhibitory activity of osmotin towards *S. cerevisiae* [34]. This may provide an alternative explanation for the increased resistance of *A. fumigatus*, *S. cerevisiae* and *P. digitatum* towards PAF26 compared to *N. crassa* that is highly sensitive to PAF26.

Remarkably, PAF96 labeled the cell envelopes in 100% of the cells of all the fungi analyzed and thus the cationic domain (PAF96 possesses only this domain) is involved in the interaction of PAF26 with this cell component. However, in *N. crassa* only, a cationic interaction is not essential for internalization because PAF95 (which lacks the cationic domain) is still internalized. Double labeling of TMR-PAF96 with the cell wall stain CFW indicated that PAF96 possibly associates with either the inner part of the thickened cell wall or at the plasma membrane (Figure 3B). This demonstrates that the peptide is at least able to diffuse through the cell wall. These observations, in conjunction with the lack of antifungal activity of PAF96, demonstrate that PAF26 does not exert its antifungal killing action at the cell surface, although the cell surface might be required for the interaction and thus antifungal activity as recently characterized in the antifungal plant defensin NaD1 [35]. PAF96 lacks the tryptophan-phenylalanine-tryptophan C-terminal tail of PAF26. The importance of tryptophan residues for the antibacterial activity of AMPs has been recognized [36] and they have been shown to facilitate the interaction with model micelles [37]. Recently, addition of tryptophan/phenylalanine motifs to short and cationic AMPs was shown to enhance bacterial killing [38], and also increase selectivity by lowering the toxicity to human epithelial and red blood cells [39]. PAF26 has been previously demonstrated to cause unusually low hemolysis and toxicity to human cells in cell culture [23]. Our work reveals that tryptophan residues are also involved in the internalization of this class of AMPs by fungal cells. In the previous alanine scan experiment with PAF26, the substitution of phenylalanine at position 5 (see Table 1) by alanine had only a minimal effect on antifungal activity [26]. This result indicates that phenylalanine is not likely to have a major role in peptide internalization/activity, and therefore points towards tryptophan being the critical residue required for cell penetration of PAF26 into fungal cells. Failure to enter microbial cells has been related to the lack of antimicrobial activity for other inactive AMP analogs such as derivatives of buforin II in *E. coli* [13], histatin 5 in *C. albicans* [40] and Sub5 in *A. nidulans* [41]. Only Sub5 contains tryptophan residues. It was shown that the amino acid substitutions that disrupted Sub5 antifungal activity involved tryptophan as well as arginine, lysine and valine residues, and therefore it was not possible to assign the observed phenotype to any specific amino acid change.

Although membrane lytic mechanisms are recognized as the primary mode-of-action for the majority of AMPs, increasing evidence supports the existence of AMPs, which exert an array of multifactorial effects including other specific interactions with the cell envelope as well as intracellular targets [5], [7]–[10]. In a previous study a transcriptomic approach was used with the model yeast *S. cerevisiae* to characterize the mode-of-action of PAF26 in comparison with the cytolytic peptide Melittin [24]. In the present study, we determined the sub-cellular location of PAF26 using high resolution confocal microscopy of three representative *S. cerevisiae* knockout strains previously identified as exhibiting increased resistance or sensitivity to PAF26 (Table 2). Our analyses correlated the percentage of cells in the cell population exhibiting complete cell labeling by TMR-PAF26 with differences in the sensitivity of the Δ*arg1* and Δ*ecm33* mutants to PAF26 (Figure 7). They also revealed that the disruption of *ARG1* is linked to increased PAF26 accumulation in intracellular vacuolar-like organelles which correlates with increased resistance to the antifungal activity of the peptide. Arg1p is a cytosolic enzyme, fundamental to the biosynthesis or arginine, metabolism/recycling of amino groups and the urea cycle. Moreover, blockage of the endogenous NO production derived from arginine was related to the PAF26-resistant phenotype of the Δ*arg1* yeast mutant [25]. Interestingly, the inactive PAF95 does not contain arginine residues and was trapped within vacuoles of *N. crassa*, wherein it was not toxic to fungal cells. Analyses of the kinetics of internalization of PAF26 [17] and histatin 5 [15], [42] indicated that the active transport from vacuoles to the cytosol is concomitant with cell killing. In the proline-rich AMP apidaecin, the internalization by bacteria and their killing were also separated and uptake into cells was necessary but not sufficient for antimicrobial activity of selected analogs [12]. From the available data we can hypothesize that containment of PAF26 and PAF95 within vacuoles preserves fungal cells from the peptide's toxicity and that the metabolism of arginine is involved in the transport of cationic peptides from vacuoles to the cytoplasm. Significantly, cationic residues are important not only for the electrostatic attraction and interaction with the fungal cell envelope but also for intracellular killing mediated by PAF26 transport from the vacuole into the cytosol.

As part of this report, we have demonstrated for the first time the activity and cell penetrating properties of the rationally designed peptide PAF26 against the human pathogen *A. fumigatus* and then extended our previous results with other model and economically important fungi [21], [23], [24]. Acute invasive pulmonary aspergillosis caused by *A. fumigatus* is a rapidly progressive and frequently lethal infection in immunocompromised patients [43]–[45]. However, existing treatments are limited to only a small number of antifungal drugs, such as azoles, echinocandins or polyenes [46], [47]. The identification of AMPs as promising pharmaceutical agents against this pathogen is currently being investigated [48]–[51]. Previous reports have demonstrated antifungal activity of AMPs against *A. fumigatus* in model animal pathosystems [50] and therefore this class of antifungal agents holds promise for the future treatment of aspergillosis. Different AMPs have also been assayed against the related model species *A. nidulans* [41]. In the present study, we found that the antifungal activity of PAF26 inhibits conidial germination in *A. fumigatus* (Figure 4A) at low micromolar concentrations, similar to that reported previously for other fungi [17]. PAF26 is in the lower size range of peptides that are active against this fungus although the acylated palmitoyl-Lys-Ala-DAla-Lys tetrapeptide has also been reported to be active against *A. fumigatus* [50]. The only previous examples of antifungal peptides that have amino acid sequence similarity to PAF26 are the larger Sub5 [41] and hLF(1–11) [49], which are cationic and contain two and one tryptophan residues, respectively.

The transport of PAF26 to the cytoplasm from the vacuoles of the *A. fumigatus* cells induced the disappearance of the H1-GFP fluorescence indicating the dissolution of nuclei, which occurred just before or was coincident with cell death (Figures 4B and 5). At no point was co-localization of H1-GFP and TMR-PAF26 observed indicating that PAF26 does not become specifically recruited to the nucleus as previously reported for the Tat (47–58) CP-AMP [19]. This is consistent with our observations that correlate killing with the active transport of peptide out of the vacuoles into the cytoplasm [17].

The novel findings described here can presumably be extended to other AMPs with similar physicochemical properties and functionalities as we have previously proposed [17]. It remains to be determined whether the C-terminal hydrophobic domain of PAF26 can direct the internalization into fungal cells of other unrelated peptidic or even not-peptidic motifs. Future experiments will address this issue as well as the relevance of using PAF26 as a model for studies on other cationic AMPs.

## Materials and Methods

### Fungal strains and growth conditions

Microorganisms used in this work were: *Neurospora crassa* WT 74-OR23-1VA *matA* strain (obtained from the Fungal Genetic Stock Center, FGSC#2489), *Aspergillus fumigatus* CEA10 strain (FGSC#1163) and a derivative with the histone H1 tagged with GFP (PGpdA-H1-sGFP) were kindly provided by Drs. Joanne Wong Sak Hoi and Jean Paul Latgé (Pasteur Institute, Paris), *Penicillium digitatum* strain PHI26 (CECT 20796) [52], and strains of *Saccharomyces cerevisiae* listed in Table 2. *Neurospora crassa* and *A. fumigatus* strains were grown in standard liquid Vogel's medium [53] at 35°C and 37°C respectively, and *P. digitatum* on potato dextrose agar (PDA) (Difco-BD Diagnostics, Sparks, MD) plates at 24°C until the culture conidiated. Conidia were collected and quantified with a hemocytometer to adjust to concentrations of 6.5×10^4^ conidia/ml for *N. crassa* and 1×10^6^ conidia/ml for *A. fumigatus* in 10% Vogel's liquid media, and to 2.5×10^4^ conidia/ml for *P. digitatum* in 5% potato dextrose broth (PDB) (Difco-BD Diagnostics) media used in the antifungal assays. *S. cerevisiae* strains were grown in YPD (yeast peptone dextrose) plates and liquid media.

### Antifungal peptides

PAF26, PAF95 and PAF96 peptides (Table 1) were purchased from GenScript (GenScript Coorp., NJ, USA) at ≥95% purity, and were synthesized by solid-phase methods using *N*-(9-fluorenyl)methoxycarbonyl (Fmoc) chemistry. Peptides were also synthesized labeled with tetramethyl-rhodamine (TMR-PAF26, TMR-PAF95 and TMR-PAF96) by covalent modification of their N-terminus. Stock solutions of PAF26 and PAF96 peptides were prepared at 1–5 mM in 5 mM 3-(*N*-morpholino)-propanesulfonic acid (MOPS, Sigma), pH 7 buffer and stored at −20°C. PAF95 and TMR-labeled peptides were firstly dissolved in a small volume of dimethylsulfoside (DMSO) according to the manufacturer's guidelines and then diluted to ∼200 µM in MOPS, to keep the DMSO concentration at less than 1% in the stock solution.

### Dyes and chemicals

Fluorescent dyes included the cell death marker propidium iodide (PI) (Sigma, MO, USA), the vacuolar marker Oregon Green 488 carboxylic acid (cDFFDA) (Molecular Probes, OR, USA) and the chitin binding dye calcofluor white (CFW) (Sigma). Stock solutions were made in DMSO or water as appropriate. Working concentrations used in live-cell imaging experiments were 5 µg/ml for PI, 20 µM cDFFDA and 50–100 µg/ml for CFW.

### *In vitro* antifungal activity assays

Fungistatic activity against *N. crassa* and *A. fumigatus* was microscopically analyzed by quantifying the percentage of germination (germ tube formation) after 3 h at 35°C for *N. crassa*, and after 4–5 h at 37°C for *A. fumigatus* exposed to the different peptide concentrations (see next section). Fungistatic activity against *P. digitatum* was assayed in 96-well plates as described [22]. Fungicidal activity of peptides towards conidia of *N. crassa* and *A. fumigatus* was determined by incubating peptide-treated conidia with the cell death marker PI, and analysis by wide-field fluorescence microscopy. Three replicas were analyzed for each treatment. A minimum of 100 conidia were analyzed for each peptide concentration in each replicate. Living and dead cells were discriminated from each other using the PI fluorescence, as described before [17]. Fungicidal activity against *S. cerevisiae* strains was assayed as described [24] by incubation of serial 5-fold dilutions from 1×10^6^ CFU/ml exponentially grown cells treated with a 64 µM concentration of each peptide for 24 h, and subsequently plating onto YPD peptide-free plates. All antifungal activity experiments were repeated at least three times. Statistical analyses of the distribution of PAF26 across populations of *S. cerevisiae* strains were carried out with the software package StatGraphics Plus 5.1 (StatPoint, Hemdon, VA). If necessary, data were log transformed to fulfill the equal variance criteria of the ANOVA tests.

### Live-cell fluorescence microscopy

Techniques of live-cell imaging at high resolution optimized for filamentous fungi have been carried out following previously described methodologies [54], [31], [32]. Time-lapse confocal laser scanning microscopy was performed using a Radiance 2100 system (Bio-Rad Microscience, Hemel Hempstead, UK) which was mounted on a Nikon TE2000U Eclipse inverted microscope equipped with blue diode, argon ion and HeNe lasers. Excitation and emission wavelengths were as follows. Excitation at 405 nm and emission at 420 nm (long pass filter) to visualize CFW fluorescence, excitation at 488 nm and emission at 515–530 nm (bandwidth filter) to visualize cDFFDA fluorescence, and excitation at 543 nm and emission at 580–700 nm (bandwidth filter) to visualize TMR-peptides and PI fluorescence. Simultaneous brightfield images were captured with a transmitted light detector. Where appropriate, Kalman filtering (*n* = 1) was used to improve the signal-to-noise ratio of individual images. Laser intensity and exposure was kept to a minimum to reduce photobeaching and phototoxic effects. Imaging was carried out a room temperature. Confocal images were captured using Lasersharp software (v. 5.1, BioRad Microscience) and were subsequently processed using ImageJ software (v. 1.44, MacBiophotonics, Canada).

Wide-field epifluorescence imaging was also performed with an inverted Nikon Eclipse TE2000E microscope (Nikon, Kingston-Upon-Thames, UK) equipped with a DXM1200F camera and the ACT-1 software for image acquisition. Propidium iodide fluorescence was excited at 550 nm using a CoolLED pE excitation system (CoolLED Ltd., Hampshire, UK). A 575 nm long-pass emission filter was used for visualization.

Live-cell imaging was performed with cells incubated in an 8-well slide culture chambers (Nalge Nunc International, Rochester, NY). The culture chamber was mounted on the inverted microscope.

## Supporting Information

Figure S1 **Representative confocal microscopy images from the data set of experiments shown in** **Figure 2A** **.** Localization of 5 µM of the different TMR-labeled PAF peptides in *N. crassa* conidia 1 h after incubation with the peptides. The percentage of cells with different patterns of peptide localization are shown in Figure 2A.
(TIF)

Figure S2 **Dose-response curves of the fungistatic effects of PAF peptides on** ***P. digitatum*** **.** Conidia were incubated with the peptides PAF26 (black circles), PAF95 (white triangles) and PAF96 (white squares) in 5% PDB at 24°C in microtiter plates. Curves show the mean OD (492 nm) ± standard deviations of three replicate samples after 72 h of incubation. Other experimental details as described [22].
(TIF)

Movie S1 **Time-lapse confocal microscopy of TMR-PAF26 internalization, uptake into vacuoles and associated vacuolar expansion in conidia and conidial germlings of** ***A. fumigatus*** **.** In the left panel the movie shows merged fluorescence images for TMR-PAF26 (in red) and H1-GFP labeled nuclei (in green). In the right panel the movie shows the same cells imaged with brightfield optics. The peptide was applied at a concentration of 5 µM and images are shown from 5 min after peptide addition. The time course was recorded at room temperature with frames captured at 4.8 sec intervals over the period of 25 min.
(AVI)
