# Supplementary figures and images for: Two Functional Motifs Define the Interaction, Internalization and Toxicity of the Cell-Penetrating Antifungal Peptide PAF26 on Fungal Cells
Source: PLoS One. 2013 Jan 21;8(1):e54813. doi: 10.1371/journal.pone.0054813 (PMC3549957; doi:10.1371/journal.pone.0054813)

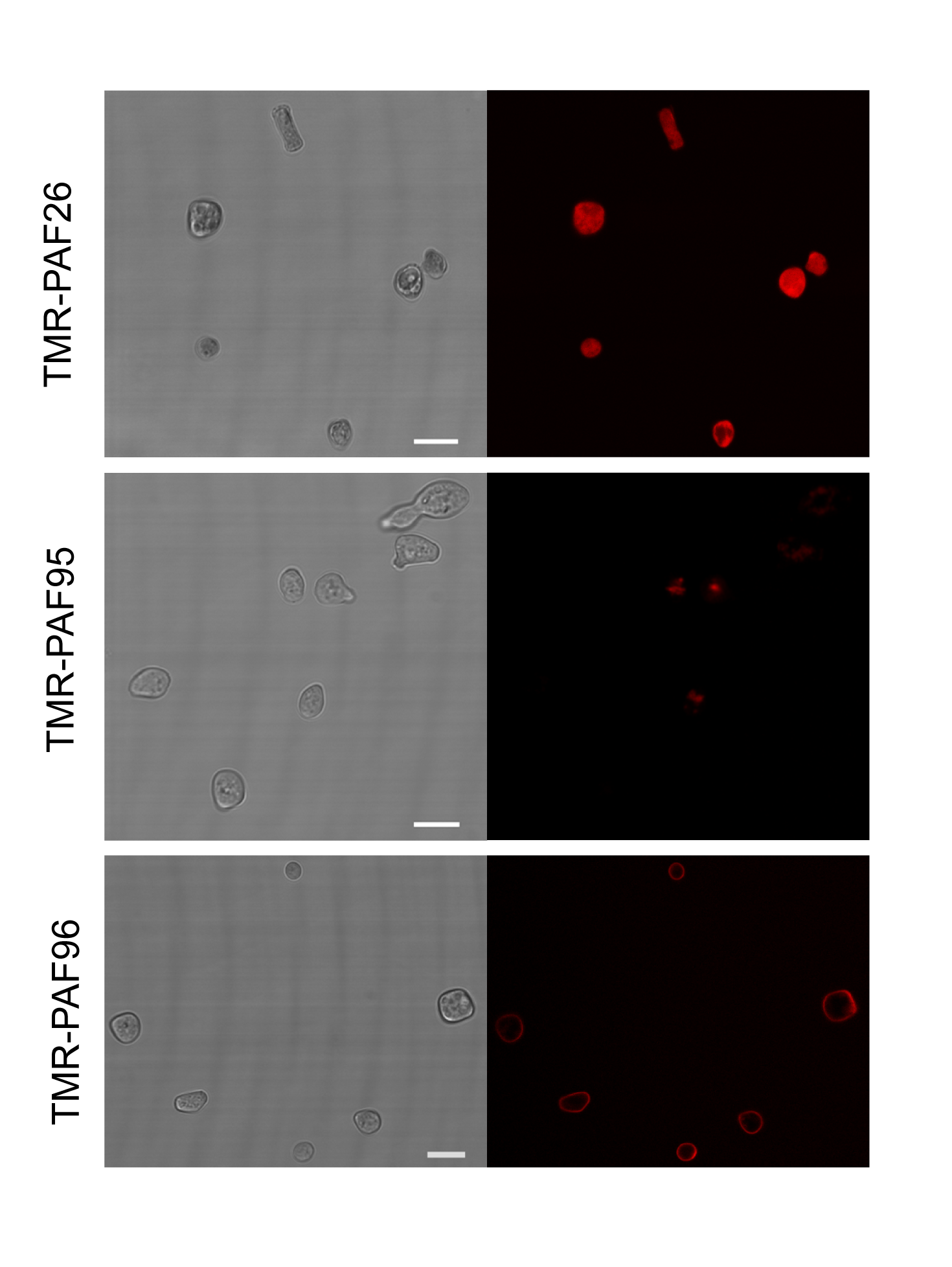

Supplement: Figure S1 — Representative confocal microscopy images from the data set of experiments shown in Figure 2A . Localization of 5 µM of the different TMR-labeled PAF peptides in N. crassa conidia 1 h after incubation with the peptides. The percentage of cells with different patterns of peptide localization are shown in Figure 2A. (TIF) [file pone.0054813.s001.tif]

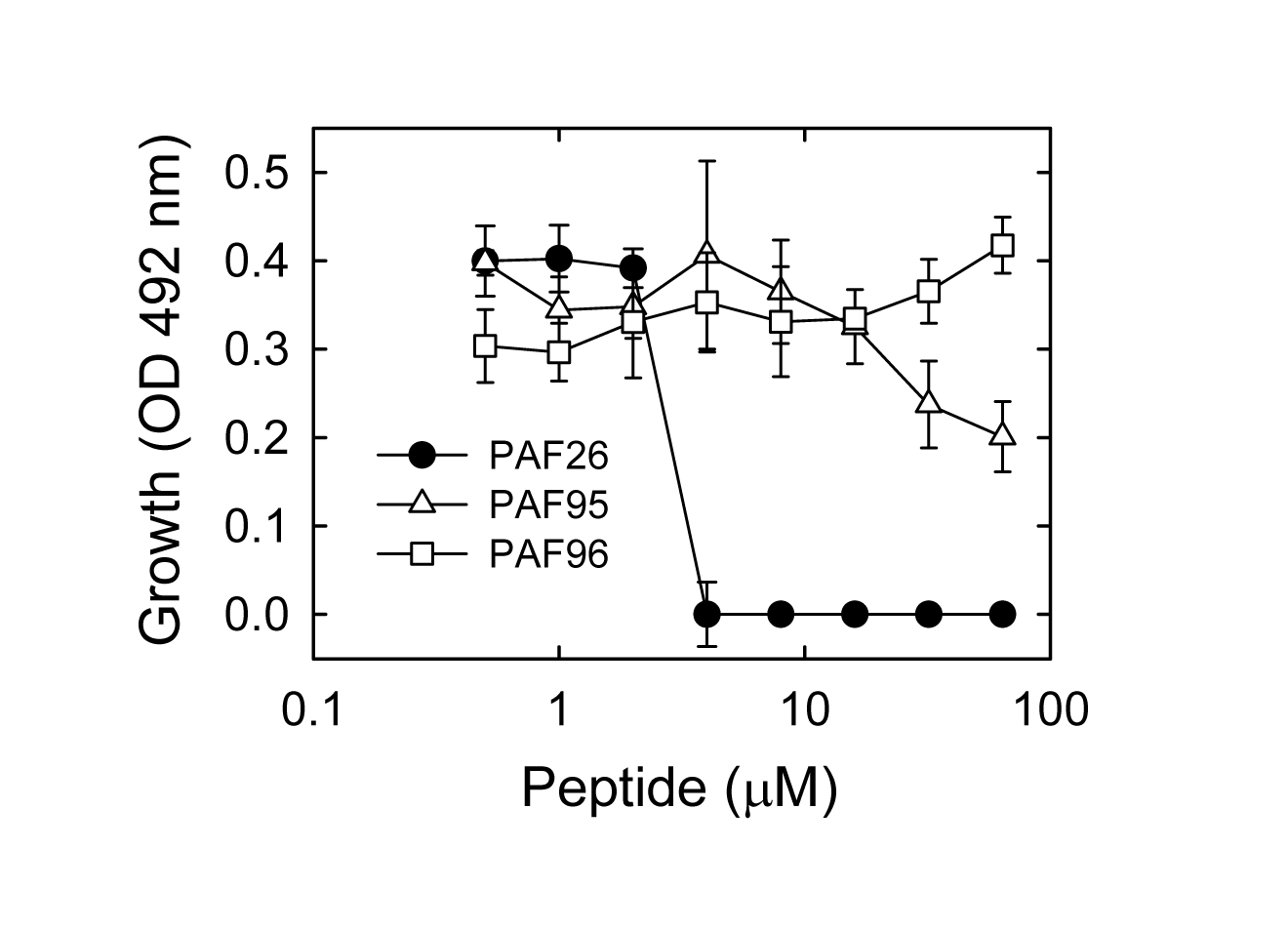

Supplement: Figure S2 — Dose-response curves of the fungistatic effects of PAF peptides on P. digitatum . Conidia were incubated with the peptides PAF26 (black circles), PAF95 (white triangles) and PAF96 (white squares) in 5% PDB at 24°C in microtiter plates. Curves show the mean OD (492 nm) ± standard deviations of three replicate samples after 72 h of incubation. Other experimental details as described [22]. (TIF) [file pone.0054813.s002.tif]
